# Commitment, partnerships and operational research: three priorities for 11 EMR countries to achieve TB elimination

**DOI:** 10.5588/ijtldopen.23.0470

**Published:** 2024-01-01

**Authors:** M. van den Boom, K. Bennani, G. B. Migliori, L. D’Ambrosio, R. Centis, A. Parvez Sayed, H. Y. Atta, Y. Hutin

**Affiliations:** ^1^ World Health Organization, Regional Office for the Eastern Mediterranean Region, Cairo, Egypt;; ^2^ Servizio di Epidemiologia Clinica delle Malattie Respiratorie, Istituti Clinici Scientifici Maugeri, Istituto di Ricovero e Cura a Carattere Scientifico, Tradate, Italy;; ^3^ Public Health Consulting Group, Lugano, Switzerland

**Keywords:** TB, TB elimination, TB elimination preparedness assessment

## Abstract

**BACKGROUND:**

In 2022, 11 of 22 Member States of the WHO Eastern Mediterranean Region (EMR) had an estimated TB incidence of <20 cases per 100,000 population. We assessed preparedness for elimination and provided recommendations to pursue the process.

**METHODS:**

We surveyed 11 EMR national TB programme managers and collected information on eight TB elimination framework domains using a close-ended data collection tool. We compiled, consolidated and validated data, including a virtual consultation before triangulating data with other sources.

**RESULTS:**

Implementation was sufficient (≥74%) for 5 of 8 domains, highest for TB infection management, TB preventive treatment, laboratory service, drug management, drug-resistant TB and TB-HIV collaboration (89%, 83% and 78%, respectively). Countries ranked lowest for commitment (73%), operational research and infection control (63%), and partnership/collaborations (41%). Five countries reached >80% when consolidating the responses, reaching sufficient from all domains. Two reached <50%.

**CONCLUSION:**

Key identified obstacles to TB elimination in EMR were insufficient commitment/financing, sub-optimal partnerships/collaborations and operational research calling for 1) all-stakeholder-inclusive, sustainably funded TB elimination plans, 2) cost-effective tools to exchange strategic information and build operational research capacity, and 3) improved collaboration.

## RÉSUMÉ

**CONTEXTE:**

En 2022, 11 des 22 États membres de la Région de la Méditerranée orientale de l'OMS avaient une incidence de la TB estimée à moins de 20 cas pour 100 000 habitants. Nous avons évalué l'état de préparation à l'élimination et formulé des recommandations pour poursuivre le processus.

**MÉTHODES:**

Nous avons interrogé 11 responsables de programmes nationaux de lutte contre la TB dans la région de la Méditerranée orientale et recueilli des informations sur huit domaines du cadre d'élimination de la TB à l'aide d'un outil de collecte de données à questions fermées. Nous avons compilé, consolidé et validé les données, y compris lors d'une consultation virtuelle, avant de les trianguler avec d'autres sources.

**RÉSULTATS:**

La mise en œuvre était suffisante (≥74%) pour 5 des 8 domaines, les plus élevés étant la gestion de l'infection tuberculeuse, le traitement préventif de la TB, les services de laboratoire, la gestion des médicaments, la TB pharmacorésistante et la collaboration TB-VIH (89%, 83% et 78%, respectivement). Les pays se sont classés au dernier rang pour l'engagement (73%), la recherche opérationnelle et la lutte contre l'infection (63%) et le partenariat/la collaboration (41%). Cinq pays ont atteint >80% lors de la consolidation des réponses, atteignant un niveau suffisant dans tous les domaines. Deux pays ont atteint un taux de réponse inférieur à 50%.

**CONCLUSION:**

Les principaux obstacles à l'élimination de la TB dans les pays de l'Union européenne sont un engagement/un financement insuffisant, des partenariats/collaborations sous-optimaux et une recherche opérationnelle nécessitant 1) des plans d'élimination de la TB incluant toutes les parties prenantes et bénéficiant d'un financement durable, 2) des outils rentables permettant d'échanger des informations stratégiques et de renforcer les capacités de recherche opérationnelle, et 3) une meilleure collaboration.

TB elimination is the ultimate expression of the End TB Strategy. In 1990, the Wolfheze Initiative, a think tank promoted by the WHO, the KNCV Tuberculosis Foundation and the International Union Against TB and Lung Diseases analysed the possibility of eliminating TB in low TB incidence countries of Europe, North America and Asia.^1,2^ Subsequently in 2014, scientists from the European Respiratory Society (ERS), European Centre for Disease Prevention and Control (ECDC) and the WHO used a survey to describe the level of preparedness for TB elimination in Europe, focusing on countries with an incidence of <20 cases/100,000 population.^3^ The WHO used the conceptual framework of this survey to define TB elimination,^4^ and this 2014 WHO framework still guides low-incidence countries to progress towards elimination.^4^ The ECDC^5^ and the Pan American Health Organization^6^ published regional documents stipulating that prevention and strong governance were prerequisites for TB elimination. However, as of 2023, the WHO had not yet developed specific plans or approaches aimed at eliminating TB in a region or sub-region.

**Figure 1.**
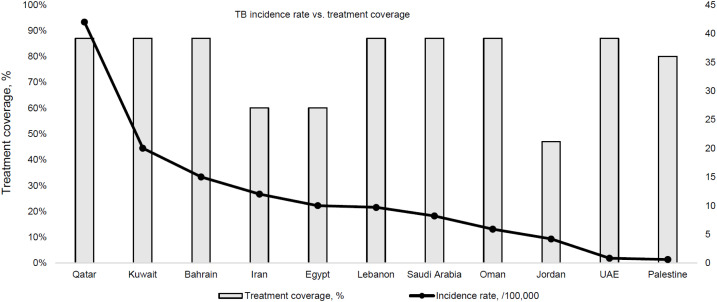
Estimated TB incidence vs. treatment coverage in 11 of 22 countries of WHO Eastern Mediterranean Region, 2022.

To date, the WHO Eastern Mediterranean Region (EMR) did not achieve the milestones and targets of reducing TB incidence and mortality called for in the WHO End TB Strategy.^7^ The region is heterogeneous in terms of TB. EMR was home to 9.9% of the world’s population in 2022,^8^ and accounted for 8.1% of the global estimated TB incidence.^9^ In EMR, 42% of the estimated cases in 2022 were missed, but treatment success rate of drug-susceptible TB exceeded 90%. Less than 15% of the estimated multidrug-resistant TB (MDR-TB) cases were diagnosed with less than 13% of those undergoing treatment. However, in 2022, despite these major regional challenges, the WHO estimated that 11 of 22 EMR countries had a TB incidence of <20 cases/100,000,^7^ with varying treatment coverage (Figure 1). These countries were committed to pursuing TB elimination. This group of countries comprised Bahrain, Egypt, Islamic Republic of Iran, Jordan, Kingdom of Saudi Arabia, Kuwait, Lebanon, Oman, Palestine and United Arab Emirates (UAE), plus Qatar (which, although above the defined threshold, reported a very low TB incidence and high treatment coverage in the native-born population). The Syrian Arab Republic was not included despite being formally slightly under 20 cases/100,000: the rationale for exclusion was that, in view of its specific context and following prolonged armed conflict and associated worsening of TB risk factors, the actual TB incidence was expected to be much higher and above the pre-elimination or elimination thresholds.

Supporting countries towards elimination spearheads the efforts to end TB in EMR. Therefore, the TB programme of the WHO EMR (RTB) designed a survey to assess the level of preparedness of these 11 low-incidence EMR countries, and to inform a region-specific TB elimination approach. This survey was based on the eight specific areas defined in the WHO TB Elimination Framework.^4^ This paper summarises and discusses the results of the survey and analyses its implications towards reaching TB elimination in the EMR.

## METHODS

### Definitions

The WHO defines ‘TB elimination’ as fewer than one notified TB case per million population per year, and considers this threshold sufficiently low to ensure that TB would not re-emerge as a public health threat.^4^ Similarly, the WHO defines ‘pre-elimination’ as <10 cases notified per million population per year.^4^ Other relevant definitions employed are those included in the 2022 WHO Global Tuberculosis Report.^7^

### Survey population

The survey population consisted of 11 national TB programme (NTP) managers of the WHO EMR.

### Design

This was a cross sectional survey.

### Data collection

#### Data collection instrument

The close-ended data collection instrument included items relevant for programmatic implementation of a regional TB elimination approach (electronic Annex): TB infection, laboratory services, drug resistance, data collection, quality of care, commitment, research/infection prevention and control (IPC), partnership. RTB developed a colour code to allow NTPs to self-evaluate the current status for each item featured in the data collection instrument. Red denoted absence of a given activity or plan, yellow indicated that the activity or plan was at the draft or initial stage, or was undergoing the approval process (intermediate), while green meant that the given activity or plan was sufficient or sufficiently implemented. As this study did not involve human subjects, no ethical approval was needed.

#### Data collection procedures

Following pre-test and revisions that involved three EMR countries between December 2021 and January 2022, RTB requested the NTP managers of each country to complete the internet-based data collection instrument in February 2022. RTB compiled and consolidated the input into the self-evaluation conducted by the NTP managers.

### Data validation

RTB presented the initial consolidated results to the representatives of the 11 countries surveyed during the EMR regional TB elimination consultation held in March 2022. The objectives of the consultation were to validate the findings and define next steps for the design of an EMR-specific TB elimination approach. Country representatives validated their input during this event, and provided suggestions, which we captured in April 2022. We ensured the results were consistent with the data included in the 2022 WHO Global TB Report.^7^ National TB managers validated the resolution of all discrepancies.

### Data analysis

First, we examined each indicator of each domain for each country with sufficient, intermediate and absent status in order to identify patterns that would lead to recommendations, domain by domain. Second, within each domain and for all countries, we calculated the proportion of each indicator with sufficient, intermediate and absent status using the total number of responses as denominator. Third, overall for each domain, we aggregated all indicators for all countries to calculate the proportion of indicators with sufficient, intermediate and absent status using the total number of responses as denominator.

## RESULTS

### Overview

**Figure 2.**
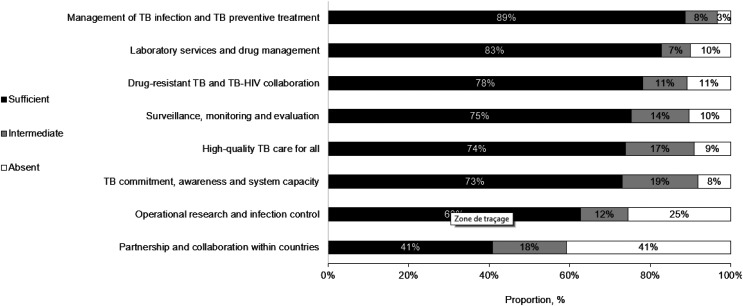
Readiness for TB elimination by domain, 11 countries of the WHO Eastern Mediterranean Region, 2022.

**Figure 3.**
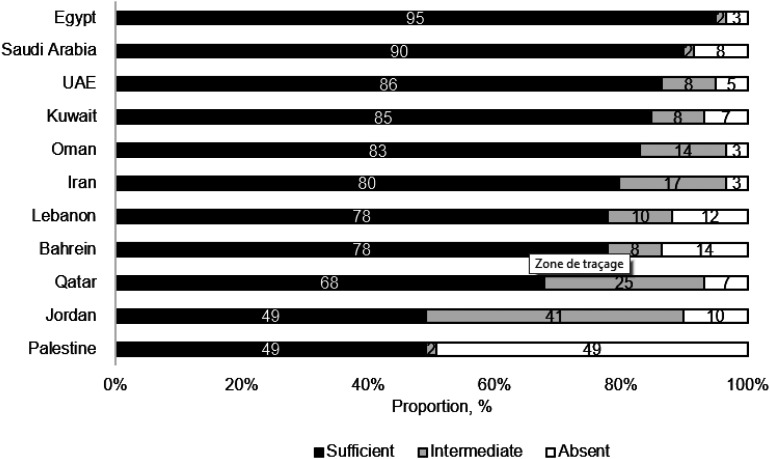
Readiness for TB elimination according to eight domains by country, 11 countries of the WHO Eastern Mediterranean Region, 2022. UAE = United Arab Emirates.

Eleven low TB incidence countries from the EMR participated. Overall, sufficient implementation was equal to or exceeded 74% for 5/8 domains (Figure 2), while ‘commitment’, ‘operational research and infection control’ and ‘partnership and collaboration within countries’ were less well implemented, at respectively 73%, 63% and 41%. Five countries reached more than 80% when consolidating the responses of sufficient from all domains (Figure 3).

### Commitment, awareness and capacity of health systems

All countries reported having a TB programme, and 10 countries engaged the private sector. Ten countries reported TB reference centres and engaged professional societies. Eight countries indicated the existence of a strategic plan, with seven having guidelines for elimination and five involving non-governmental organisations (NGOs) and the civil society. The indicators most often reported as insufficient were those about the availability of budget estimates and financing.

### Surveillance, monitoring and evaluation

One country reported sufficient implementation across the entire domain. All countries indicated that surveillance data were regularly reported and analysed. Ten countries had specific national targets for TB control and elimination. No countries had plans for future regular epidemiological trend modelling involving risk groups.

### Laboratory services and drug management

Four countries reported sufficient implementation in this domain. As one key WHO had recommended rapid TB diagnostic molecular testing, all countries had implemented GeneXpert (Cepheid, Sunnyvale, CA, USA), and 10 had a referral system for bacteriological specimens. Similarly, 10 countries had first- and second-line drugs available, while three experienced localised and partial stock-out of medicines. Five countries reported sub-optimal implementation of new rapid diagnostics other than GeneXpert.

### High-quality care for all

Three countries only reported sufficient implementation across the entire domain. All countries reported that TB care was provided free for all and having special strategies or policies in place to care for the vulnerable and risk groups. Four countries had adequate national poverty targeting programmes or people supporting patients on treatment (treatment supporters).

### Drug resistance and TB-HIV collaborative activities

Four countries reported sufficient implementation under this domain. All countries indicated existing collaboration between TB and HIV programmes. Six provided integrated TB-HIV care services. Nine countries had reference centres or specialised hospitals for DR-TB management. Three countries had no (or insufficient) implementation of DR-TB treatment adherence-facilitating and social support schemes.

### Management of infection and preventive treatment

Countries reported best performance/scored highest in this domain out of all the eight surveyed. All countries reported conducting TB infection (TBI) screening, contact tracing and provision of preventive treatment. Five countries indicated that the capacity to monitor TBI treatment completion was lacking. Three lacked updated TBI screening and treatment guidance.

### Operational research and infection control

Overall, countries performed better on TBI control than on operational research. Whereas 10 countries indicated having implemented infection control measures, 3 had specific budgets for conducting operational research and 4 claimed full engagement in conducting operational research.

### Partnership and collaboration within countries

Countries faced the greatest challenge in this domain. Eight countries reported to have a strategic collaboration/partnerships with other countries in TB elimination, and eight lacked a national ‘TB Consilium’, a professional platform where complex TB cases could be peer-discussed and clinically managed.^10^

## DISCUSSION

The 11 EMR countries surveyed differed in terms of preparedness for TB elimination. Countries reported doing well in the management of infection, prevention, laboratory services and drug management. They struggled most for three domains that were commitment, operational research and partnership /collaborations.

Regarding commitment, countries involved professional health societies to work towards elimination. These engagements were not always supported by sufficient budgets or financing. Limited domestic financial investments were available to ensure that national control and elimination plans were fully funded. This gap prevented further progress towards elimination.^11^ Within the health sector, effective TB elimination plans have ambitious and specific objectives that require full implementation to reach the target. Treatment success rate of drug-susceptible TB should exceed 95%. There is a need for effective TB infection management by 2030, consisting of key interventions such as screening 90% of individuals belonging to risk groups, achieving at least 90% completion rate for the individuals undergoing TBI treatment and implementing TBI registers. Outside of the health sector, there is insufficient realisation that TB is a social disease. TB is associated with poverty. Support is needed aside of medical treatment.^12,13^ Nevertheless, the absence of treatment supporters hinders further progress towards TB elimination, and most countries do not sufficiently benefit from or collaborate with national poverty targeting programmes. Countries striving to approach TB elimination need to prioritise sustainable approaches to address the root causes of poverty in all geographical areas and population groups.^14^ In EMR, Lebanon^15^ and Jordan^16^ reported good practice on country-specific TB elimination plans. These include cost estimates and budget lines, in addition to technical guidance on TB elimination.

Performance in operational research was the second lowest. Several elements suggest that limited budgets to support research plans and operational research focal points hindered progress towards TB elimination. Operational research is also neglected because it requires very specialised capacity at planning and implementation stages, which therefore takes up considerable human resources. Such resources are often prioritised differently because of a preference for short- and medium-term return on investment. The WHO TB e-learning platform advances national capacities at different levels effectively and have high outreach potential.^17^ It can enhance research endeavours to improve the likelihood of TB elimination. Insights gained from digital outreach experiences during the COVID-19 pandemic could prove valuable for the efficient deployment and utilisation of digital tools at the national level.

Strategic in-country and international partnerships/collaborations was the third major gap objectified by this survey. Such arrangements have benefits for TB elimination. Involving the civil society and other non-state stakeholders can help towards elimination as this complements national programme activities along the entire cascade of TB care.^18^ Community players, for example, can help in case-finding and holding, improving the probability of reaching favourable treatment outcomes. Many countries reported the absence of a ‘TB Consilium’, which can be an in-person or online/virtual platform to discuss complex or challenging TB patient management within a national team of experts. Such consilium has also the potential to increase outreach to and support coverage of hard-to-reach patients, for example, in rural areas or vulnerable groups, such as the Extension for Community Healthcare Outcomes (ECHO) Project.^19^ A national TB Consilium can also link to ‘international TB Consilia’, thereby taking advantage of clinical opinions from international experts.^10^ These activities build inter and intra-country capacity and facilitate learning. Furthermore, participation in various international TB elimination research projects can strengthen national capacity. Within the Global Tuberculosis Network (GTN),^20^ intensified exchanges of project results between EMR TB WHO Collaborating Centres could accelerate countries’ progress towards TB elimination. The survey pointed further to additional weaknesses in individual, case-based surveillance, drug management, TB specimen referral systems and patient-centred approaches. These issues could be addressed by improving digital data systems, pooling drug management and referrals, and further integrating TB services.

Our survey suffers from several limitations. First, variability may have affected results, as questions may have been interpreted differently. This could have happened over time by the same respondent, or through different respondents in the same country. This may have impacted accuracy and reproducibility. Second, respondents may have been inclined to answer rather more positively than self-critically because of their national ownership and accountability within the in-built limitations of the tool used (questionnaire). We repeated interactions with respondents and complemented the survey by the final workshop to validate the data and triangulate them with the data included in the 2022 WHO Global TB Report^7^ to limit this potential bias.

This analysis leads to three main conclusions. First, political commitment for TB is insufficiently backed by budgets that benefit from domestic funding. Second, operational research suffers from resource limitations and competing priorities. Third, opportunities to rally partnerships and collaborations that would better involve non-state actors, such as civil society organisations, are sometimes missed. On the basis of these conclusions, we can propose a number of recommendations. First, programmes should involve all TB stakeholders to develop or update national strategic plans that include TB elimination components. These plans must be funded from increased, sustainable domestic sources that should come from the health system and through a more convincing demonstration of benefits yielded by TB multisectoral work, brought about by the TB multisectoral accountability framework (MAF-TB). Second, countries should design and introduce self-sustained, cost-effective tools, including digital ones, to exchange strategic information and build operational research capacity. Third, countries should collaborate more domestically and internationally. At the country level, this could be done through clinical and public health consultation platforms, such as country-level and cross-national ‘TB consilia’^10^ and WHO Collaborating Centres. Internationally, countries could analyse their practices and exchange their experience through the RTB-led MAF-TB and country support work. Overall, EMR countries can progress towards TB elimination by 2030 guided by this analysis. Elimination has the potential to inspire and guide higher TB burden countries through cross-fertilisation of ideas. Ultimately, the adoption of TB elimination efforts should drive the EMR TB Regional Action Plan 2023–2030^21^ from the apex.
